# Resource allocation for depression management in general practice: A simple data-based filter model

**DOI:** 10.1371/journal.pone.0246728

**Published:** 2021-02-19

**Authors:** Breanne Hobden, Mariko Carey, Rob Sanson-Fisher, Andrew Searles, Christopher Oldmeadow, Allison Boyes

**Affiliations:** 1 Health Behaviour Research Collaborative, School of Medicine and Public Health, Faculty of Health and Medicine, University of Newcastle, Callaghan, NSW, Australia; 2 Priority Research Centre for Health Behaviour, University of Newcastle, Callaghan, NSW, Australia; 3 Hunter Medical Research Institute, New Lambton Heights, NSW, Australia; University of Hong Kong, HONG KONG

## Abstract

**Background:**

This study aimed to illustrate the potential utility of a simple filter model in understanding the patient outcome and cost-effectiveness implications for depression interventions in primary care.

**Methods:**

Modelling of hypothetical intervention scenarios during different stages of the treatment pathway was conducted.

**Results:**

Three scenarios were developed for depression related to increasing detection, treatment response and treatment uptake. The incremental costs, incremental number of successes (i.e., depression remission) and the incremental costs-effectiveness ratio (ICER) were calculated. In the modelled scenarios, increasing provider treatment response resulted in the greatest number of incremental successes above baseline, however, it was also associated with the greatest ICER. Increasing detection rates was associated with the second greatest increase to incremental successes above baseline and had the lowest ICER.

**Conclusions:**

The authors recommend utility of the filter model to guide the identification of areas where policy stakeholders and/or researchers should invest their efforts in depression management.

## Introduction

Depression is the most prevalent mental health illness worldwide and has demonstrated a 50% increase in incident cases from 1990–2017 [1]. Depression is among the leading causes of disease burden worldwide and is associated with a high economic burden [2–5]. Depression also impacts quality of life, as it has been associated with unemployment, adverse impacts on personal relationships, economic disadvantage and homelessness [6–8].

In many developed countries, primary care plays a central role in the management of depression [9–11]. The role of primary care is multifaceted and includes diagnosis, management and/ or referral to specialist mental health professionals such as psychiatrists, psychologists or clinical social workers. Given the important role of primary care practitioners in provision of mental health care, there is significant interest in developing ways to improve outcomes for people with depression within this setting. While there are evidence-based treatments for depression, there may be barriers which prevent those in need from receiving the care needed to optimise outcomes. Therefore, there is potential to improve outcomes by implementing strategies to improve care at specific points in the treatment trajectory. The key points in the care pathway include increasing the proportion: 1) of people who are accurately identified as depressed by their doctor; 2) who are offered evidence-based treatments; and 3) of patients who adhere to the recommended treatment.

A meta-analysis indicated that only 47% of people who are depressed are recognised as such by their general practitioner (GP) [12]. Similarly, our study involving over 1,500 Australian primary care patients found that GPs identified only 51% of patients with elevated depression scores on the Patient Health Questionnaire (9-items) as having clinically significant depressive symptoms [13]. Several factors may also impede GPs in offering evidence-based treatments to people with depression. Providers commonly report barriers related to inadequate skills to manage depression, as well as the time required to provide intensive psychological therapies [14–16]. When evidence-based treatments are offered, the effectiveness of such treatments may be hindered by poor patient adherence. Patients with depression have been found to be more likely to be non-adherent to prescribed treatments than those who are not depressed [17–20].

With increasing competition for the health dollar, there is interest among policy stakeholders in identifying priority research and investment areas. While complex mathematical modelling is typically the norm in decisional analytics [21], the level of sophistication required to use such methods may be prohibitive for decision or policy-makers and researchers without access to expertise [22]. A simplified decisional tool that could guide the identification of areas where policy stakeholders and/or researchers should invest their efforts seems warranted. Such a tool was developed by members of the research team [23, 24]. The model is derived from a decision tree concept, using a logic modelling approach to filter data through the sequence of steps associated with treatment outcomes in healthcare settings. The relationship to a decision tree is based on the filter model’s use of cost and outcome at key junctures. The model can be used to compare costs and patient outcomes for two or more scenarios. The application of the model in this paper is intended to highlight how the tool could be applied to compare the effectiveness and cost-effectiveness of intervention strategies for improving depression outcomes in primary care.

### Aim

To illustrate the potential utility of a simple filter model in understanding the patient outcome and cost effectiveness implications for depression interventions in primary care.

## Materials and methods

The development of the model has been described in detail elsewhere [23, 24]. The model is targeted at users without specialised economics or statistical skills or knowledge. It is underpinned by theoretical and modelling principles of decision trees and cost-effectiveness analysis to create a decisional analytic framework. Modelling is conducted using an Excel spreadsheet with built-in calculations for a series of steps. The user will follow the steps as outlined below.

### Steps 1–3: Identify the relevant group data

1. **Population**: define and quantify the population of interest (e.g., general population, certain age cohorts);
2. **Target group**: define and quantify the target of the intervention (e.g., depressed individuals);
3. **Setting attendance**: enter the proportion of the target group attending the setting of interest (e.g., depressed individuals attending primary care);

### Steps 4–6: Adding the filters representing different intervention scenarios

When the user enters the relevant proportions for the following steps, the number of persons at each level in the model is calculated and used as output in the subsequent step. For example, the calculated number of persons of the target group attending the setting of interest who are detected for the condition of interest (step 4) are used as the starting figure for reach and adherence (step 5).

4 **Detection**: the proportion of the target group attending the setting of interest who are detected for the condition of interest. An intervention can be applied here to increase detection using relevant intervention effect (increase in proportion detected) and cost estimates.
5 **Reach and adherence:** the following are estimated by the user. An intervention can be applied at each of these steps with the relevant intervention effect (increase reach or adherence proportions) and cost estimates required.
  - Reach: The proportion of individuals with the condition of interest who are offered treatment by their healthcare provider;
  - Adherence: The proportion of individuals with the condition of interest who would comply with the offered treatment;
6 **Effect on outcome:** for a dichotomous outcome measure of treatment success, specify the expected proportion of those that are offered the intervention that will achieve a successful outcome following the intervention.

The outputs derived from the above data are outlined in **Table 1**.

**Table 1:** Model outputs and calculations used to derive outputs.

Outcome Data	Calculation		
Cost per participant exposed to the intervention	(Total cost of the intervention(s) under a scenario across all targeted patients) (Number of patients who are offered the intervention)		
Cost per successful outcome	(Total cost of the intervention(s) under a scenario across all targeted patients) (Number of patients who achieve a successful outcome)		
Incremental cost compared to baseline	(Total cost of detection and intervention across all patients in one scenario)	-	(Total cost of detection and intervention across all patients for baseline or usual care)
Incremental number of successes compared to baseline	(Number of patients who achieve a successful outcome under a scenario)	-	(Number under baseline or usual care)
Incremental cost effectiveness ratio (ICER)^1^	(Incremental cost compared to baseline). (Incremental number of successes compared to baseline)		
Policy advice	The model provides a summary of whether each scenario is more or less expensive (in terms of the incremental cost) and more or less effective (in terms of the number of successful outcomes) compared to baseline or usual care.		

^1^The ICER is the ratio of the change in cost to the change in effectiveness between each scenario and usual care. This metric can be used to compare the relative cost-effectiveness of different alternatives against the cost and consequences of usual care.

### Data analytic procedures: Application of the filter model to depression treatment

To demonstrate the potential utility of the decision analytical filter model for improving depression outcomes, data for four scenarios, including ‘usual care’, and three hypothetical conditions, are presented. A successful outcome for the following scenarios was defined as an individual reaching ‘remission’, i.e., below threshold depression levels. The baseline scenario attempted to model standard care in the identification and treatment of depression for primary care patients, using data drawn from the literature (see **Table 2**). This scenario assumed no interventions were implemented and that usual care was provided. The three hypothetical scenarios represent an improvement above usual care in each of the filters. An estimation of the intervention effect and relevant costs associated with achieving these improvements were entered into the model. While efforts were made to draw these estimates from the literature, the aim of the model, in its current status, is to illustrate hypothetical scenarios. Further, the population and target group can be adjusted for different countries, regions or settings. The Australian population has been used in the model presented for illustrative purposes, however, the derived data is drawn from international references. Filter one, i.e., detection, examined an intervention aimed at increasing detection of depression in primary care. This involved administering a free validated depression measure to primary care patients electronically using a touchscreen computer upon presentation to their appointment. Filter two, i.e., reach, involved increasing the offer of evidence-based depression treatment from providers. This intervention is based on training and support for GPs in appropriate management of depression. Filter three, i.e., adherence consisted of increasing patient adherence to offered treatment. This involved a telephone intervention to monitor the progress of patients undergoing treatment for depression and provide adherence strategies. All filters were assumed to operate independently from one another, for example an increase in the proportion of patients detected for depression did not increase the proportion of patients offered treatment by GPs. Dollar amounts are presented in Australian Dollars (AUD).

**Table 2:** Model parameters and output under usual care and three hypothetical intervention scenarios.

Step	Definition	Proportion	Unit Cost of intervention	No. of persons
(1) Population	*Australian population aged 18-75yrs*	n/a		15,055,403
(2) Target group	*Current depression* ^a^	8.9%		1,339,931
(3) Opportunity for Detection	*Proportion of target group attending GP at least once per annum* ^b^	81%		1,085,344
(4) Detection	*Proportion detected as at risk*			
	Baseline: (no intervention) ^c^	47%	$0	510,112
	Filter 1: (**intervention: implementation of routine screening for depression**) ^d^	77%	$5	835,715
	Filter 2: (no change to baseline) ^c^	47%	$0	510,112
	Filter 3: (no change to baseline) ^c^	47%	$0	510,112
(5) i. Reach	*Proportion detected who were offered treatment*			
	Baseline: (no intervention) ^e^	28%	$0	142,831
	Filter 1: (no change to baseline) ^e^	28%	$0	234,000
	**Filter 2: (GP intervention: education and training)** ^f^	69%	$247	351,977
	Filter 3: (no change to baseline) ^e^	28%	$0	142,831
(5) ii. Adherence	*Proportion detected*, *offered treatment who are adherent*			
	Baseline: (no intervention) ^g^	51%	$0	72,844
	Filter 1: (no change to baseline) ^g^	51%	$0	119,340
	Filter 2: (no change to baseline) ^g^	51%	$0	179,508
	**Filter 3: (Patient intervention: telephone follow-ups)** ^h^	65%	$98	92,840
(6) Effect on outcome	*Proportion achieving remission at 6 months follow-up*			
	Baseline: (no intervention) ^i^	37%	$0	26,952
	Filter 1: (no change to baseline) ^i^	37%	$0	44,156
	Filter 2: (no change to baseline) ^i^	37%	$0	66,418
	Filter 3: (no change to baseline) ^i^	37%	$0	34,351
**MODEL OUTCOMES**				
**Cost/exposure**	*Cost per participant exposed to intervention*			
	Baseline:	$0		
	Filter 1:	$5		
	Filter 2:	$247		
	Filter 3:	$98		
**Cost/outcome**	*Cost per successful outcome*			
	Baseline:	$0		
	Filter 1:	$95		
	Filter 2:	$1,309		
	Filter 3:	$265		
**Incremental cost**	*Incremental cost compared to baseline*			
	Filter 1:	$4,178,574		
	Filter 2:	$86,938,336		
	Filter 3:	$9,098,352		
**Incremental number successfully treated**	*Incremental number of successes compared to baseline*			
	Filter 1:	17,204		
	Filter 2:	39,466		
	Filter 3:	7,399		
**ICER**	*Incremental cost effectiveness ratio*			
	Filter 1:	$243		
	Filter 2:	$2,203		
	Filter 3:	$1,230		

Active intervention components at relevant steps are bolded.

## Results

**Table 2** presents the data inputs for each of the steps used in the model’s hypothetical scenarios. The literature used to inform the filters are presented as footnotes in **Table 3**.

**Table 3:** References and explanation for data used in the model (footnotes refer to those used in Table 2).

Footnote and description	Reference and notes
(a) Depression prev	• ABS 2015 [25].
(b) Size of target group attending GP	• ABS 2013 [26]: Data is for the proportion of the Australian population aged 15 years and over who attend a GP at least once annually.
(c) Detection: baseline	• GP detection of depression was assumed to be 47% based on detection rates reported by Mitchell (2009) [12].
(d) Detection: intervention	• An increase in detection of 30% (from 47% to 77%) was based on studies indicating the sensitivity of the PHQ-9 screening test to be 88% accurate in detecting depression [27]. This increase in detection was slightly reduced to account for false positives.
	• For an iPad waiting room screening intervention, a cost of $5 per person screened was estimated.
(e) Reach: baseline	• Offer of treatment was assumed to be 28% based on findings reported by Rost (2001) [28].
(f) Reach: intervention	• A 41% (from 28% to 69%) improvement rate for offering treatment was used based on intervention data reported by Rost, 2001[28] for training and educating GPs in depression management.
	• Costing for the intervention to improve treatment provisions was based on Pyne, 2003 [29] which indicated that implementation and training costs of the GP training intervention were $247 per person.
(g) Adherence: Baseline	• Adherence to depression treatment was assumed to be 51% based on findings reported by Dietrich (2004) [30].
(h) Adherence: Intervention	• Dietrich 2004 [30] found a 14% (from 51% to 65%) improvement in ≥1 follow-up appointments at six-months follow-up. While this study did not directly measure adherence to a particular treatment, attending additional follow-ups was used as a proxy measure for this.
	• Simon 2009 [31] estimated a telephone management intervention project to cost $98 per patient.
(i) Treatment effectiveness	• Six-month remission rates were derived from Dietrich 2004 [30], which found the intervention group to have a 37% remission rate. As remission was derived from those who adhered to treatment in step (f), the intervention group statistics were used for this filter.

**Table 2** highlights the findings of the hypothetical interventions applied in the filter model for managing depression in primary care. All three filters were found to be both more effective and more expensive than baseline. Filter one, involving a waiting room intervention to increase detection rates for depressed patients, resulted in an estimated increase of 17,204 patients reaching remission at six-months follow-up (compared to baseline), at an estimated cost of $95 per successful outcome. Filter two, consisting of an intervention to educate and train GPs in better management of depression, resulted in an estimated increase of 39,466 patients with depression reaching remission at six-months follow-up (compared to baseline) and cost $1,309 per successful outcome. Filter three, involving a telephone intervention to improve patient adherence to depression treatment, resulted in an estimated increase of 7,399 patients with depression reaching remission at six-months follow-up (compared to baseline) and costs $265 per successful outcome.

When examining incremental costs of each filter (as compared to baseline), filter one was the least expensive filter option with an incremental cost of $4,178,574 and an ICER of $243. Filter two was the most expensive filter with an incremental cost of $86,938,336 and an ICER of $2,203.

## Discussion

Primary care presents a unique opportunity to identify and assist individuals who may be experiencing depression. Nevertheless, scarcity in funding for service delivery requires careful consideration of where to invest research funds and healthcare budgets. The simple filter model used in this paper can help to inform decisions behind such allocation to reduce reliance on opinions or assumptions of decision-makers. The utility of the model in its application to hypothetical interventions for depression in primary care highlights its potential usefulness.

### Comparison with existing literature

Interestingly, the findings of the model in the example above indicated that filter one, which aimed to increase detection through a screening intervention using touchscreen computer tablets, was the most cost-effective filter. While there is some contention regarding screening for depression in primary care [32–34], this method of intervening represents a relatively low cost and low maintenance strategy while greatly increasing the number of successes in the hypothetical scenarios. Further, the considerable investment placed on technological health advancements [35], including mobile health [36, 37], could provide opportunities to explore remote screening options via smartphone messages and apps that link in with primary care medical records. This approach could be conducted automatically and notify healthcare providers if their patients are at-risk of depression, which would further reduce hardware and personnel time associated with in-clinic screening. Electronic screening options therefore present a feasible intervention option to improve administration rates of depression treatment. While interventions relating to reach and adherence are important areas to examine, the greater level of intensity required to undertake these interventions, as well as a smaller proportion of patients accessed, resulted in much higher ICERs for these interventions. As previously stated, the findings presented from the model are intended to be illustrative, however, where possible, the data were drawn from the literature. Therefore, the approach of targeting and comparing these different filters within primary care still warrant consideration.

### Strengths and limitations

Use of the model should be considered in light of its limitations. Firstly, the information derived from the model is only as strong as the input data provided. It is therefore limited by any inaccuracies or biases that exist in the studies the data is derived from. Further, some data used in the model were based on research which was more than ten years old. This was due to the model requiring specific information to meet the parameters, such as intervention costs per person, relevant targeted interventions (e.g., provider training) and binary study outcomes (e.g., remission rates). While this limited the data available for use, the parameters of cost and outcome are of high importance to policy and health services decision makers. This limitation is also a strength of the model, as outcomes and costs for a single intervention can also be compared when there is uncertainty in key parameters. Nevertheless, future decision-makers utilising the model to allocate large amounts of resources may consider applying quality checklists to included studies, such as the risk of bias criteria from the Effective Practice of Care (EPOC) collaboration [38] to ensure relevant information can be drawn from high quality research trials.

Additionally the model assumes that intervening at one filter does not alter rates at other filters. For instance, in the worked example, training GPs in depression management may increase the quality of the information provided to patients or improve the likelihood of GP follow-up, which may inadvertently increase patient adherence to treatment. Furthermore, incorporating the complexity of certain conditions in to this simple model is difficult. For example, depression is associated with different levels of severity and treatment pathways differ based on this severity. The presented model only accounted for intervention-related costs and did not include health care costs associated with severity of illness and hence, increased treatment intensity, healthcare provider time, nor the patient perspective in estimating costs. Severity of conditions and health care costs could be considered in future applications of the model. The model is also not able to account for inaccuracy of detection, such as false positives and false negatives and therefore issues such as unnecessary treatment and intervention were not considered. A further strength of the model is its ability to let the user enter a range of values for key input parameters. Hence, the user can vary the value of any of the filter model’s input parameters that might be associated with meaningful levels of uncertainty. This then produces a range of outcome values (or boundaries) that can be considered in decision making.

### Implications for research and/or practice

This theoretical filter model, developed using economic cost-effectiveness principles, has the potential to influence discussions on resource allocation involving decision or policy-makers for depression management in primary care. The simplicity and accessibility of the filter model represent the strengths of this approach. It is stored within a Microsoft Excel file and can be used by individuals without specialised skills, including those working within relevant care settings for depression. While the model can be applied at a national level, as was demonstrated in this paper, it could also be used to make decisions at a local level such as within a singular health services. Further, using this tool to examine depression could be expanded beyond the primary care setting to specialised mental health or hospital settings. Such an application of the tool could be used to examine the ICER of different aspects of treatment being offered by these services. The calculator is cost-free which makes it a feasible option for health care facilities to optimise cost-effective treatment outcomes.

Input data required for the tool should be evidence-based regarding target populations, reach and effectiveness. Generally, these data are likely to be available in the scientific literature and population health reports. However, another useful aspect of this model is that it highlights gaps in the current literature that prevent examining certain aspects of selected care pathways. For instance, when considering the depression literature examined, there was a lack of economic analysis reported for intervention studies undertaken. While the effectiveness of interventions in improving depression outcomes is of great importance, the cost-effectiveness is equally valuable for determining the feasibility of implementing research findings in to actual care. Future research examining depression interventions in primary care should carefully consider this aspect of research.

## Conclusions

This study highlights the utility of a simple and free filter model to conduct cost-effectiveness analysis for depression management in primary care, without complex software or specialised statistical or economic skills. Given the substantial economic burden of depression and ongoing competition for limited health care resources, there is a need to focus on strategies that optimise depression outcomes. The use of evidence-based information to assist with decision-making in these circumstances is an important undertaking. The authors recommend utility of the filter model to individual health services, researchers and decision-makings at a policy or funding level, to help inform future strategies for the management of depression in healthcare settings.

## References

[1] Liu Q, Hea H, Yanga J, Feng X, Zhao F, Lyu J. Changes in the global burden of depression from 1990 to 2017: Findings from the Global Burden of Disease study. Journal of Psychiatric Research. 2020;126:134–40. 10.1016/j.jpsychires.2019.08.002 31439359

[2] LaMontagne A, Sanderson K, Cocker F. Estimating the economic benefits of eliminating job strain as a risk factor for depression Carlton: Victorian Heath Promotion Foundation (VicHealth); 2010. 10.1097/JOM.0000000000000908 28045792

[3] Ferrari AJ, Charlson FJ, Norman RE, Patten SB, Freedman G, Murray CJL, et al Burden of depressive disorders by country, sex, age, and year: Findings from the Global Burden of Disease Study 2010. PloS Med. 2013;10(1): e1001547. 24223526 10.1371/journal.pmed.1001547 PMC3818162

[4] World Health Organization. Depression and Other Common Mental Disorders: Global Health Estimates. Geneva; 2017. Contract No.: Licence: CC BY-NC-SA 3.0 IGO.

[5] Konig H, Konig H-H, Konnopka A. The excess costs of depression: a systematic review and meta-analysis. Epidemiology and Psychiatric Sciences. 2020;29(e30):1–16. 10.1017/S2045796019000180 PMC8061284 30947759

[6] Australian Bureau of Statistics. Prevalence of mental disorders. Canberra: ABS; 2008.

[7] Cho Y, Lee JK, Kim D-H, Park J-H, Choi M, Kim H-J, et al Factors associated with quality of life in patients with depression: A nationwide population-based study. PloS ONE. 2019;14(7):e0219455 10.1371/journal.pone.0219455 31295291 PMC6623963

[8] Barile JP, Pruitt AS, Parker JL. A latent class analysis of self‐identified reasons for experiencing homelessness: Opportunities for prevention. Journal of Communit & Applied Social Psychology. 2018;28(2):94–107.

[9] Cuijpers P, Straten Av, Schaik Av, Andersson G. Psychological treatment of depression in primary care: a meta-analysis. British Journal of General Practice. 2009;59(559):e51–e60. 10.3399/bjgp09X395139 PMC2629842 19192368

[10] Bortolotti B, Menchetti M, Bellini F, Montaguti MB, Berardi D. Psychological interventions for major depression in primary care: a meta-analytic review of randomized controlled trials. General Hospital Psychiatry. 2008;30(4):293–302. 10.1016/j.genhosppsych.2008.04.001 18585531

[11] Cuijpers P, Andersson G, Donker T, Straten Av. Psychological treatment of depression: Results of a series of meta-analyses. Nordic Journal of Psychiatry. 2011;65(6):354–64. 10.3109/08039488.2011.596570 21770842

[12] Mitchell AJ, Vaze A, Rao S. Clinical diagnosis of depression in primary care: a meta-analysis. Lancet. 2009;374(9690):609–19. 10.1016/S0140-6736(09)60879-5 19640579

[13] Carey M, Jones K, Meadows G, Sanson-Fisher R, D’Este C, Inder K, et al Accuracy of general practitioner unassisted detection of depression. Aust N Z J Psychiatry. 2014;48(6):571–8. 10.1177/0004867413520047 24413807 PMC4230951

[14] Richards J, Ryan P, Mccabe MP, Groom G, Hickie IB. Barriers to the effective management of depression in general practice. Aust N Z J Psychiatry. 2004;38(10):795–803. 10.1080/j.1440-1614.2004.01464.x 15369538

[15] Barley EA, Murray J, Walters P, Tylee A. Managing depression in primary care: A meta-synthesis of qualitative and quantitative research from the UK to identify barriers and facilitators. BMC Family Practice. 2011;12(47). 10.1186/1471-2296-12-47 21658214 PMC3135545

[16] Fleury M-J, Imboua A, Aubé D, Farand L, Lambert Y. General practitioners’ management of mental disorders: A rewarding practice with considerable obstacles. BMC Family Practice. 2012;13(19). 10.1186/1471-2296-13-19 22423592 PMC3355055

[17] DiMatteo M, Lepper H, Croghan T. Depression is a risk factor for noncompliance with medical treatment. Arch Intern Med. 2000;160(14):2101–7. 10.1001/archinte.160.14.2101 10904452

[18] Grenard JL, Munjas BA, Adams JL, Suttorp M, Maglione M, McGlynn EA, et al Depression and medication adherence in the treatment of chronic diseases in the United States: A meta-analysis. Journal of General Internal Medicine. 2011;26(10):1175–82. 10.1007/s11606-011-1704-y 21533823 PMC3181287

[19] Gonzalez JS, Batchelder AW, Psaros C, Safren SA. Depression and HIV/AIDS treatment nonadherence: a review and meta-analysis. Journal of Acquired Immune Deficiency Syndromes. 2011;58(2):181–7. 10.1097/QAI.0b013e31822d490a 21857529 PMC3858003

[20] Acosta F, Rodríguez L, Cabrera B. Beliefs about depression and its treatments: Associated variables and the influence of beliefs on adherence to treatment. Revista de Psiquiatría y Salud Mental. 2013;6(2):86–92. 10.1016/j.rpsm.2012.08.001 23084794

[21] Sun X, Faunce T. Decision-Analytical Modelling in Health-Care Economic Evaluations. Eur J Health Econ. 2008;9(4):313–23. 10.1007/s10198-007-0078-x 17943332

[22] Brailsford S. Overcoming the barriers to implementation of operations research simulation models in healthcare. Clin Invest Med. 2005;28(6):312 16450620

[23] Bryant J, Noble N, Sanson-Fisher R, Searles A, Oldmeadow C, Watson R, et al Where should we target our research effort? A data-based model for determining priorities for smoking cessation research and healthcare delivery in general practice. Journal of Behavioral Economics for Policy. 2018;Accepted for Publication.

[24] Sanson-Fisher RW, Noble NE, Searles AM, Deeming S, Smits RE, Oldmeadow CJ, et al A simple filter model to guide the allocation of healthcare resources for improving the treatment of depression among cancer patients. BMC Cancer. 2018;18(1):125 10.1186/s12885-018-4009-2 29402237 PMC5800015

[25] Australian Bureau of Statistics. 4364.0.55.001—National Health Survey: First Results, 2014–15: ABS; 2015 [Available from: http://www.abs.gov.au/ausstats/abs@.nsf/Lookup/by%20Subject/4364.0.55.001~2014-15~Main%20Features~Smoking~24.

[26] Australian Bureau of Statistics. 4839.0—Patient Experiences in Australia: Summary of Findings, 2012–13: ABS; 2013 [Available from: http://www.abs.gov.au/ausstats/abs@.nsf/Lookup/4839.0main+features32012-13.

[27] Kroenke K, Spitzer R, Williams J. The PHQ-9: validity of a brief depression severity measure. J Gen Intern Med. 2001;16(9):606–13. 10.1046/j.1525-1497.2001.016009606.x 11556941 PMC1495268

[28] Rost K, Nutting P, Smith J, Werner J, Duan N. Improving depression outcomes in community primary care practice. J Gen Intern Med. 2001;16(3):143–9. 10.1111/j.1525-1497.2001.00537.x 11318908 PMC1495192

[29] Pyne JM, Rost K, Zhang M, Williams DK, Smith J, Fortney J. Cost-effectiveness of a primary care depression intervention. J Gen Intern Med. 2003;18(6):432–41. 10.1046/j.1525-1497.2003.20611.x 12823650 PMC1494878

[30] Dietrich AJ, Oxman TE, Williams JW, Schulberg HC, Bruce ML, Lee PW, et al Re-engineering systems for the treatment of depression in primary care: cluster randomised controlled trial. BMJ. 2004;329(7466):602 10.1136/bmj.38219.481250.55 15345600 PMC516659

[31] Simon G, Ludman EJ, Rutter CM. Incremental benefit and cost of telephone care management and telephone psychotherapy for depression in primary care. JAMA Psychiatry. 2009;66(10):1081–9. 10.1001/archgenpsychiatry.2009.123 19805698

[32] Gilbody S. Should we screen for depression? BMJ. 2006;332:1027–30. 10.1136/bmj.332.7548.1027 16644833 PMC1450055

[33] Thombs B RC Z. Does depression screening improve depression outcomes in primary care? The BMJ. 2014;348(g1253). 10.1136/bmj.g1253 24496211

[34] Thombs BD, Coyne JC, Cuijpers P, Jonge Pd, Gilbody S, Ioannidis JPA, et al Rethinking recommendations for screening for depression in primary care. The Canadian Medical Association Journal. 2012;184(4):413–8. 10.1503/cmaj.111035 21930744 PMC3291670

[35] Schofield P, Shaw T, Pascoe M. Toward comprehensive patient-centric care by integrating digital health technology with direct clinical contact in Australia. Journal of Medical Internet Research. 2019;21(6):e12382 10.2196/12382 31165713 PMC6682300

[36] Kao C-K, Liebovitz D. Consumer mobile health apps: Current state, barriers, and future directions. Clinical Informatics in Psychiatry. 2017;9(5). 10.1016/j.pmrj.2017.02.018 28527495

[37] Wang K, Varma D, Prospero M. A systematic review of the effectiveness of mobile apps for monitoring and management of mental health symptoms or disorders. Journal of Psychiatric Research. 2018;107:73–8. 10.1016/j.jpsychires.2018.10.006 30347316

[38] Cochrane Effective Practice and Organisation of Care (EPOC). Suggested risk of bias criteria for EPOC reviews 2015 [Available from: http://epoc.cochrane.org/epoc-specific-resources-review-authors.

